# *In Vitro* Comparison of Hypericin and 5-Aminolevulinic Acid-Derived Protoporphyrin IX for Photodynamic Inactivation of Medulloblastoma Cells

**DOI:** 10.1371/journal.pone.0051974

**Published:** 2012-12-14

**Authors:** Rainer Ritz, Christian Scheidle, Susan Noell, Florian Roser, Martin Schenk, Klaus Dietz, Wolfgang S. L. Strauss

**Affiliations:** 1 Department of Neurosurgery, Eberhard Karls University Tübingen, Tübingen, Germany; 2 Department of General, Visceral and Transplant Surgery, Eberhard Karls University Tübingen, Tübingen, Germany; 3 Department of Medical Biometry, Eberhard Karls University Tübingen, Tübingen, Germany; 4 Institut für Lasertechnologien in der Medizin und Meßtechnik an der Universität Ulm, Ulm, Germany; Wayne State University School of Medicine, United States of America

## Abstract

**Background:**

Hypericin (HYP) is a naturally occurring photosensitizer. Cellular uptake and photodynamic inactivation after incubation with this photosensitizer have neither been examined in medulloblastoma cells *in vitro*, nor compared with 5-aminolevulinic acid-derived protoporphyrin IX (5-ALA-derived PpIX).

**Methods:**

In 3 medulloblastoma cell lines (D283 Med, Daoy, and D341 Med) the time- and concentration-dependent intracellular accumulation of HYP and 5-ALA-derived PpIX was analyzed by fluorescence microscopy (FM) and FACS. Photocytotoxicity was measured after illumination at 595 nm (HYP) and 635 nm (5-ALA-derived PpIX) in D283 Med cells and compared to U373 MG glioma cells.

**Results:**

All medulloblastoma cell lines exhibited concentration- and time-dependent uptake of HYP. Incubation with HYP up to 10 µM resulted in a rapid increase in fluorescence intensity, which peaked between 2 and 4 hours. 5-ALA-derived PpIX accumulation increased in D283 Med cells by 22% over baseline after 5-ALA incubation up to 1.2 mM. Photocytotoxicity of 5-ALA-derived PpIX was higher in D283 Med medulloblastoma compared to U373MG glioma. The 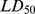 [lethal dose (light dose that is required to reduce cell survival to 50% of control)] of 5-ALA-derived PpIX was 3.8 J/cm^2^ in D283 Med cells versus 5.7 J/cm2 in U373MG glioma cells. Photocytotoxicity of HYP in D283 Med cells was determined at 2.5 µM after an incubation time of 2 h and an illumination wavelength of 595 nm. The 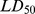 value was 0.47 J/cm^2^.

**Conclusion:**

By its 5-fold increase in fluorescence over autofluorescence levels HYP has excellent properties for tumor visualization in medulloblastomas. The high photocytotoxicity of HYP, compared to 5-ALA-derived PpIX, is convincingly demonstrated by its 8- to 13-fold lower 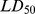. Therefore HYP might be a promising molecule for intraoperative visualization and photodynamic treatment of medulloblastomas.

## Introduction

The treatment of medulloblastomas is based on surgery and radiochemotherapy [1]. Medulloblastomas often infiltrate extremely vulnerable adjacent areas, such as the brainstem and fourth ventricle. Thus, the surgeon often faces a dilemma during resection. Knowing that the extent of resection determines the response to therapy and survival time, surgeons favor extensive or complete resection of the tumor [2]. Yet, severe and often persistent neurological deficits caused by injuries to adjacent brain structures, such as cranial nerve nuclei in the brain stem may encounter. A method of visualizing the tumor intraoperatively might improve surgical outcomes.

The introduction of fluorescence-guided resection of glioblastomas using 5-aminolevulinic acid-derived protoporphyrin IX (5-ALA-derived PpIX) has improved radicality of tumor resections and, consequently, patient outcomes [3]–[5]. Fluorescence of malignant tissue after administration of 5-ALA is caused by the accumulation of its metabolite PpIX, which is involved in the last step of heme biosynthesis – insertion of ferrous iron into PpIX by ferrochelatase forming heme. In malignant tissue, this step is impeded resulting in the accumulation of PpIX [6]. Based on results in human cell culture studies, in which glioblastoma cell lines showed significantly higher fluorescence intensity after incubation with 5-ALA as compared with neurons, we became interested in whether medulloblastoma cells also accumulates PpIX.

Our recent studies have focused on hypericin (HYP), a naturally occurring photosensitizer (PS) that is found in plants of the genus Hypericum, of which Hypericum perforatum (St. John’s wort) is the most common. HYP is a lipophilic molecule that is incorporated into the phospholipid bilayer of cell membranes and has already in the dark versatile pharmacological activities. These include antiviral, anticancer and antiangiogenic properties[7]–[11]. Takahashi et al. could show an inhibitory effect on proteinkinase C, which is involved in cell proliferation [12], [13]. Malignant gliomas have, compared to glial cells, a high proteinkinase C activity [14]. HYP has excellent properties as a PS [15]–[19]. Photodynamic therapy (PDT) is based on the generation of reactive oxygen species (ROS - either superoxide radicals, type I reaction, or singlet oxygen molecules (^1^O_2_), type II reaction) by a photosensitizer (PS) in the presence of oxygen and light. HYP has a high triplet quantum yield and a high efficiency in the formation of ROS [16], [17], [20]. The excessive production of ROS leads to oxidative stress to many biomolecules, e.g. proteins, causing cell death by induction of apoptosis, necrosis or autophagy associated cell death [21]. Semiquinone anion radicals are also important reactive intermediates in HYP mediated PDT. The site of photodamage by semiquinone anions is the quinone reducing center (Q_i_) off complex III [22]. *In vivo,* PDT causes vascular damage and effects tumor destruction additionally [23]. Efficacy of PDT *in vitro* depends on the uptake of photosensitizer and fluence rate [24]. Stylli et al. suggested that the intracellular concentration of photosensitizers in tumor tissue is a prognostic factor of therapeutic outcomes [25]. Thus, this parameter must be examined *in vitro* to develop therapeutic strategies for optimal treatment conditions.

In malignant glioma, the most frequent malignant brain tumor in adults, the value of HYP in tumor visualization and photodynamic therapy *in vitro* has been demonstrated in detail [26]. By this HYP may also be of great interest in the therapy of medulloblastomas. Based on the experience in glioblastoma, we performed this study to:

- Examine the time- and concentration-dependent fluorescence of medulloblastomas after administration of 5-ALA and HYP.
- Compare fluorescence microscopy (FM) and FACS with regard to the efficacy of measuring uptake kinetics of 5-ALA and HYP *in vitro*.
- Investigate the cytotoxicity and the photocytotoxicity as a function of photosensitizer concentration of 5-ALA and HYP in dependence of the light dose.

**Figure 1:**
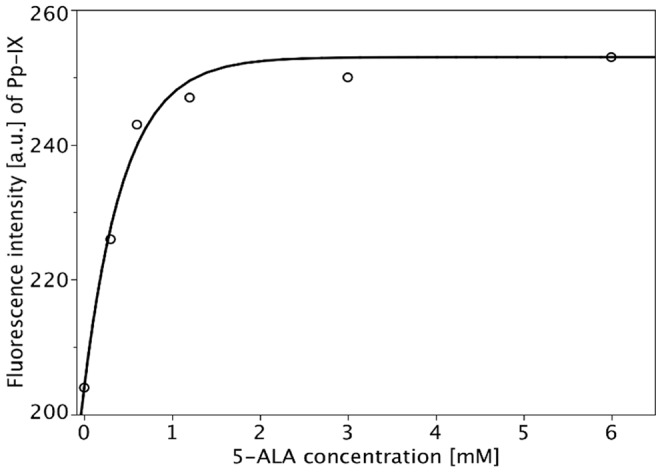
Concentration-dependent 5-ALA induction of PpIX. Each symbol represents the mean value of 60 measurements (20 individual cells/3 independent experiments) determined by fluorescence microscopy. The mathematical model used to calculate the fitted curves is given by Formula (1). The parameters are: 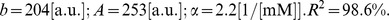

## Materials and Methods

### Photosensitizer

5-ALA was purchased from Medac (Wedel, Germany). For each experiment, fresh stock solutions were prepared by dissolving 5-ALA in PBS and adjusting to pH 7.4. Final 5-ALA concentrations ranged from 0 to 6 mM.

**Figure 2:**
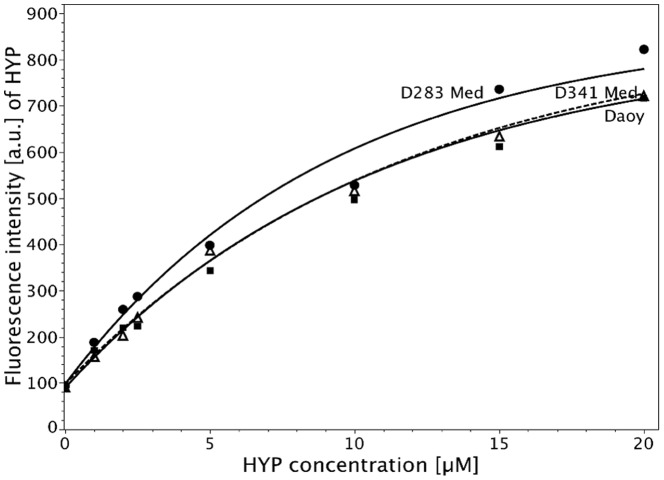
Concentration-dependent fluorescence intensity of HYP in D283 Med (dotted line), D341 Med (dashed line), and Daoy cells (continuous line) determined by fluorescence microscopy after 2 h incubation at 37°C. Mean values of the individual measurements for D283 Med, D341 Med Daoy are plotted as filled circles, filled squares, and open triangles, respectively, and the fitted curves are calculated according to Formula (1). The parameter values for 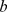, 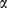, and 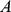 are: D283 Med: 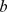 = 97.6 [a.u.], 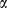 = 0.11 [1/[µM]], 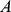 = 867.8 [a.u.]; 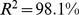; D341 Med: 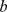 = 97.6 [a.u.], 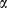 = 0.09 [1/[µM]], 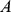 = 866.2 [a.u.]; 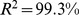; and Daoy: 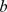 = 89.7 [a.u.], 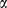 = 0.09 [1/[µM]], 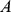 = 832.5 [a.u.]; 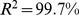.

HYP was obtained from Phytochem (Ichenhausen, Germany); the purity exceeded 99%. Stock solutions of HYP were made in DMSO at 2 mM and stored in the dark at −20°C. For all experiments, incubation media was freshly prepared in cell culture medium with a final fetal calf serum (FCS, Biochrom KG, Berlin, Germany) content of 10% for HYP and 1% for 5-ALA under sterile conditions.

**Figure 3:**
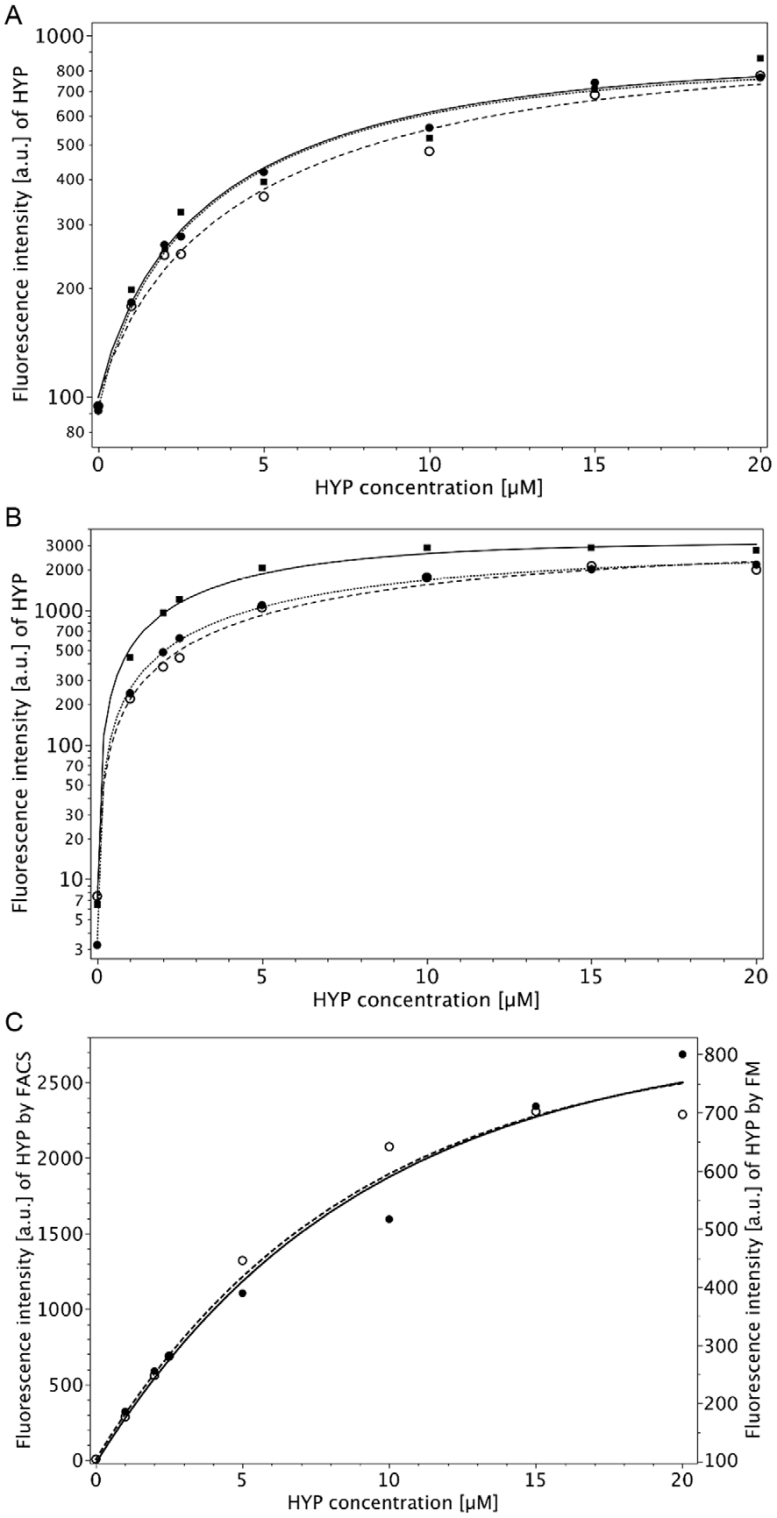
Concentration-dependent intracellular HYP accumulation in D283 Med cells determined by fluorescence microscopy (FM) (A) and FACS analysis (B) after 2h-incubation at 37°C (three independent experiments). Each symbol is the mean value of 20 cells (FM) or 200.000 cells (FACS). Comparison of FM (continuous line, right ordinate) and FACS (dashed line, left ordinate) data. (**C**) Curves are based on the means of the values in A and B. Note that the curves are nearly identical, taking into account the difference in autofluorescence. The means for 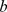, 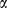, and 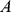 are: FM: 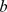 = 97.4 [a.u.], 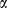 = 0.115 [1/[µM]], 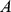 = 832.6 [a.u.], 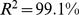, FACS: 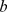 = 5.4, 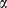 = 0.111 [1/[µM]], 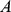 = 2775 [a.u.], 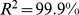. These high values for the percentage of the explained variances are due to the fact that the residuals were calculated as relative errors instead of absolute errors.

### Cell Culture

Human medulloblastoma cell lines (D283 Med, ATCC No. HTB-185; Daoy ATCC No. HTB-186; D341 Med, ATCC No. HTB-187) were obtained from American Type Culture Collection, Manassas, VA, USA. U373 MG cells were obtained from the European Collection of Cell Cultures, Wiltshire, UK, ECACC No. 890811403) and handled as reported previously [26]. Cell lines were grown in 25 ml tissue cell culture flasks (NUNC, Langenselbold, Germany) with minimal essential medium (MEM, ATCC, USA) supplemented with 10% FCS and antibiotics (penicillin (100 IU/ml), and streptomycin (100 µg/ml), both obtained from PAA Laboratories, Cölbe, Germany and maintained at 37°C and 5% carbon dioxide (CO_2_) in an incubator (Cellstar; Nunc, Wiesbaden, Germany). Cells were detached by incubation with Accutase™ (PAA Laboratories) at 37°C for 5 min after being rinsed with phosphate-buffered saline (PBS, Invitrogen, Karlsruhe, Germany). For all experiments, cells were seeded at 300 cells/mm^2^ and grown for 48 h under standard conditions (37°C/5% CO_2_).

**Figure 4:**
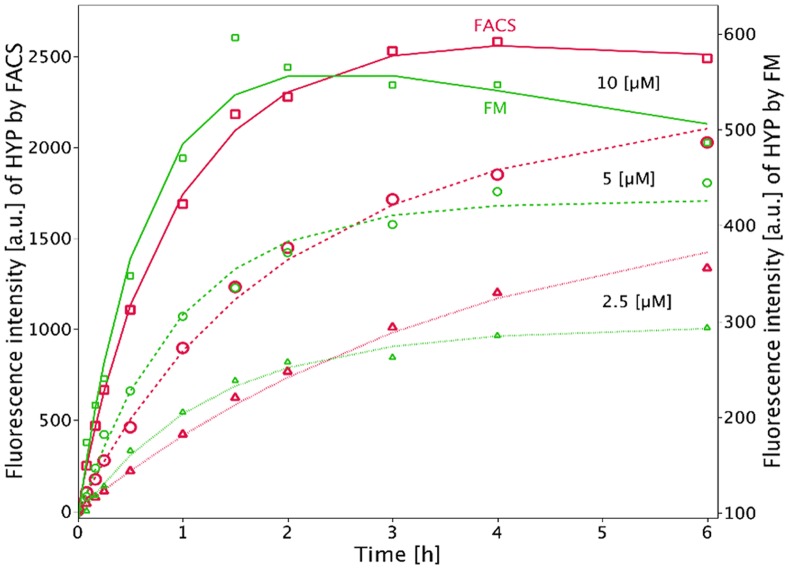
Comparison of FM (green) and FACS (red) of time-dependent HYP uptake in D283 Med cells incubated with 2.5 µM (dotted lines, triangles), 5 **µM (dashed lines, open circles), and 10** **µM (continuous lines, open squares) HYP.** Curves were fitted according to Formula (2). The means for 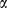, 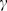, and 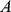 for 2.5, 5, and 10 µM, respectively, are: FACS: 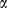: 0.168, 0.486, 1.08 [1/h]; 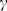: 0.048, 0, 0.036 [1/h] and 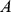: 3336, 2529, 2959 [a.u.], 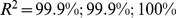 FM: 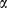: 0.96, 1.05, 1.52 [1/h], 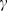: 0, 0, 0.066 [1/h] and 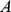: 295, 426.5, 639.5 [a.u.], 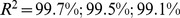. We calculated relative errors instead of absolute errors.

### Intracellular Accumulation

Intracellular accumulation of 5-ALA-derived PpIX and HYP was measured by FM (Olympus BX 61, Olympus, Hamburg, Germany) and flow cytometry (FACSSORT, Becton Dickinson, San Jose CA, USA).

**Table 1:** Comparison of time dependent HYP uptake by method.

	Mean of 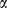 [per hour]		
*HYP concentration [µM]*	*FM*	*FACS*	*P value*
2.5	0.960	0.168	0.045
5	1.05	0.486	0.139
10	1.518	1.08	0.244

Time dependent HYP uptake was characterized for the two methods (FM and FACS) by formula 2; 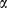 was estimated for FACS and FM analysis, taking into account Bonferroni adjustment of the p-values (z-tests) there was no significant difference between the 2 methods for concentrations of 5 and 10 µM.

The fluorescence microscope was equipped with a PLAPO60x oil immersion objective (N.A. 1.4) and the following filter sets were used: to measure 5-ALA-derived PpIX fluorescence: band pass filter 370–390 nm (excitation), dichroic mirror 440 nm, long pass filter 455 nm (emission) - AHF Analysentechnik AG, Tübingen, Germany; to measure HYP fluorescence (U-MWG2 filter - Olympus, Hamburg, Germany): band pass filter 510–550 nm (excitation), dichroic mirror 570 nm, long pass filter 570 nm (emission). Images were obtained using a charge-coupled device camera (F-View II, Olympus Soft Imaging Solutions GmbH, Münster, Germany). Fluorescence intensity was assessed in 20 individual cells by the pixel grey scale intensities of single cells with the software analySIS (Soft Imaging Systems, Münster, Germany).

**Figure 5:**
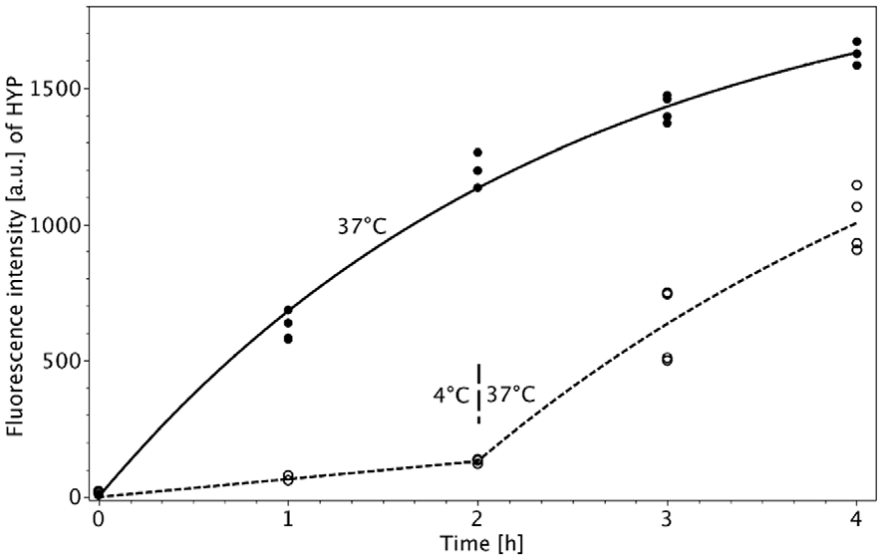
Cellular accumulation of HYP in D283 Med medulloblastoma cells (concentration 2.5 **µM) versus temperature [4°C for 2 h and subsequently 37°C for further 2 h (O) vs. 37°C for 4 h (**•**)], as measured by flow cytometry.** The observations are fitted according to the Formula (3a) (continuous line for constant 37°C incubation) and Formula (3b) (dashed line for temperature switch at 2 hours). The initial slopes are given in Table 2. (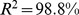; we calculated absolute errors.).

**Table 2:** Cellular HYP uptake rates 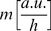
at different temperatures.

Slope	*m*	95% CI	
*m*_0_	834	750	924
*m*_1_	66	36	96
*m*_2_	552	456	654

Slope of HYP uptake at 4°C (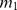), after switch from 4 to 37°C (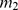) and at 37°C (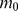) and 95% confidence intervals. 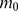 and 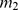 are significantly different (p<10^−4^, z-test).

For flow cytometric measurements cells were cultivated in 24-well plates (NUNC) and incubated with 5-ALA or HYP. After rinsing with PBS (500 µl) cells were detached from the plates with Accutase™ (PAA, Cölbe, Germany). Cells from 4 identically treated wells were pooled and filtered through a metal mesh (100–112-µm pore size; Haver & Boecker, Oelde, Germany). After addition of propidium iodide (Sigma-Aldrich, Steinheim, Germany) dissolved in PBS (2 mg/ml; 10 µl per 1 ml cell suspension) fluorescence of HYP and 5-ALA treated cells was measured by flow cytometry. Fluorescence was excited at 488 nm. Photosensitizer fluorescence was detected at an emission wavelength of 585+/−21 nm. To determine cell viability an emission wavelength of >670 nm was used. The data were analyzed with CellQuestTM (version 4.0.2, Becton Dickinson, Erembodegem-Aalst, Belgium) on 200,000 cells.

**Figure 6:**
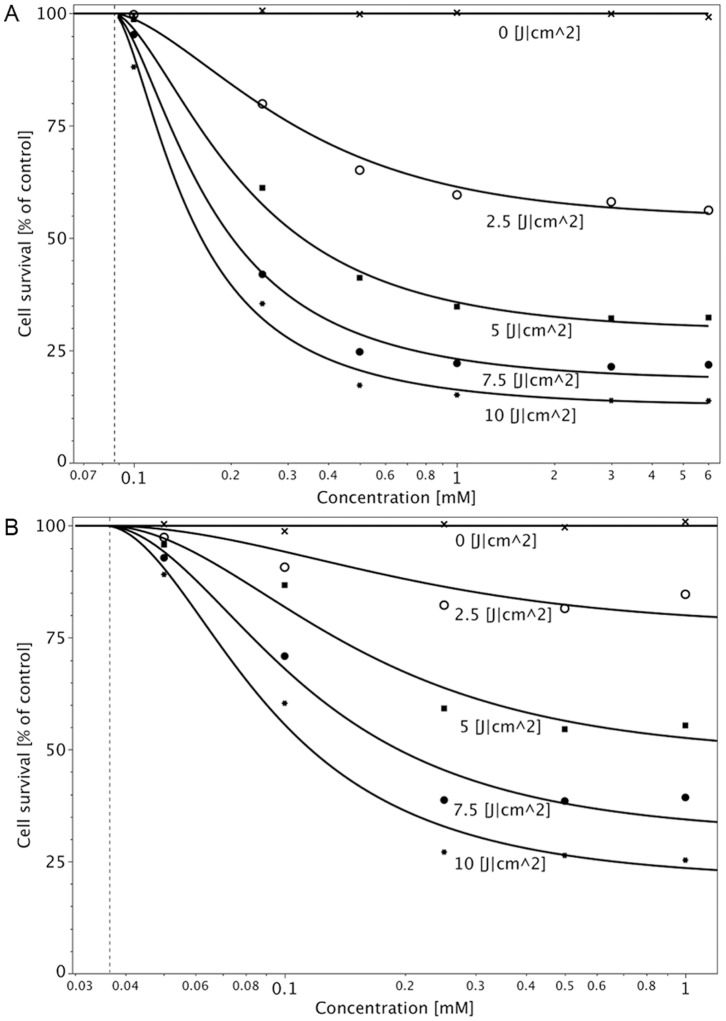
Photocytotoxicity of 5-ALA-derived PpIX in D283 Med (A) and U373 MG cells (B). Concentration- and fluence-dependent photocytotoxicity is demonstrated after incubation with 5-ALA for 2 h. Illumination was performed at 635 nm, and light was delivered at 20 mW/cm^2^ at doses of (x) 0, (o) 2.5, (▪) 5, (•) 7.5, (*) and 10 J/cm^2^. Cell survival was measured by NR assay 48 h after illumination. Cell survival was calculated per Formula (4). Dashed line shows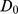. (For the two cell lines the 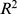 values are 99.7% (D283 Med) and 98.7% (U373 MG), respectively.)

### Cytotoxicity

To measure cytotoxicity (toxicity in the dark) of 5-ALA/5-ALA-derived PpIX and HYP, cells were grown in 24-well plates (NUNC). 3×10^4^ cells/well were seeded and incubated with different PS concentrations (5-ALA: 0–15 mM; HYP: 0–20 µM) at 37°C (5% CO_2_) for 2 h. After being rinsed with PBS cells were incubated with PS-free culture medium for 48 h (37°C/5% CO_2_). Thereafter, culture media were discarded and cells were rinsed twice with isotonic saline. Cell survival was assessed by neutral red (NR) assay. Cultures were thus incubated (37°C) with an aqueous NR solution (Biochrom, Berlin, Germany) diluted with culture medium 1∶25 resulting in final NR concentration of 0.012% and adjusted to pH 7.4) for 2–3 h (by addition of 500 µl NR solution to each well). Supernatants were discarded and cells were rinsed twice with isotone saline. NR was extracted from the cells by addition of 150 µl of a mixture of water/ethanol/acetic acid (1/1/0.02). After gentle agitation for 10 min, a 100 µL aliquot of each sample was transferred into a microtiter plate (Greiner, Frickenhausen, Germany). Absorbance was measured at 570 nm (with a reference wavelength of 690 nm) on a plate reader (Lucy 1, anthos Mikrosysteme, Krefeld, Germany) and analyzed with the anthos WinRead software (version 2.3) [26]–[28]. Cell survival was calculated as the percentage of living cells incubated with the PS versus living cells of non-incubated controls. For each cell line, at least 3 independent experiments were performed in quadruplicate, resulting in at least 12 values for each case.

**Table 3:** Comparison of 5-ALA phototoxicity of medulloblastoma vs. glioma.

Cell line	D283 Med	U373MG	
5-ALA incubation concentration 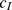 [mM]	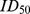 -value (95% CI) [J/cm^2^]	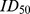 -value (95% CI) [J/cm^2^]	p-value
0.1	44.6 (36.5–58.3)	12.6 (11.7–13.7)	p<0.0001
0.25	6.5 (6.2–6.8)	5.9 (5.7–6.1)	p = 0.0039
0.5	3.8 (3.6–4.0)	5.7 (5.4–5.9)	p<0.0001
1	3.3 (3.0–3.5)	5.8 (5.6–6.0)	p<0.0001

Phototoxicity of 5-ALA in medulloblastoma cell line D283 Med compared to glioblastoma cell line U373 MG after incubation time of 2 h. 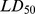-values were calculated according to formula (4), 95% confidence intervals (CI) are given.

### Photocytotoxicity

To measure the photocytotoxicity of 5-ALA-derived PpIX and HYP, cells were cultured in 4-well plates and incubated with the PS at non-cytotoxic concentrations for 2 h (5-ALA: 0–6 mM; HYP: 2.5 µM). After being rinsed with PBS and incubated with culture medium (without PS), the cells were illuminated with a dye laser (model 375, Spectra Physics, Mountain View, USA) that was pumped by an argon ion laser (model 2030, Spectra Physics).

**Figure 7:**
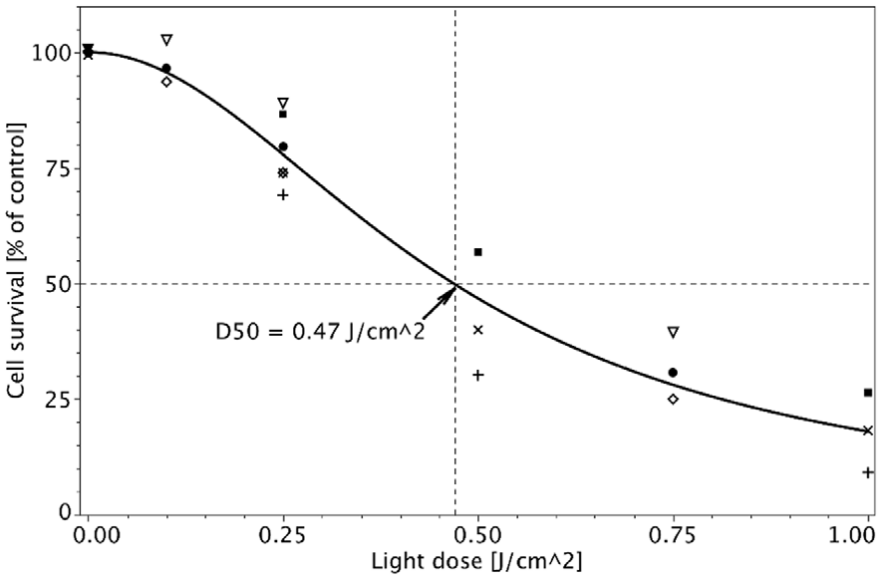
Photocytotoxicity of HYP in D283 Med cells at 2.5 **µM and an incubation time of 2 h.** Illumination was performed at 595 nm, and light was delivered at 10 mW/cm^2^ at doses of 0, 0.1, 0.25, 0.5, 0.75, and 1 J/cm^2^. ▪, •, □, ○, ◊, and △ are the mean observed values of six independent experiments (Two experiments on each of three days.). Cell survival was measured by NR assay 48 h after illumination. Cell survival was fitted by Formula (5). 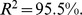 (We calculated absolute errors.).

Cells were irradiated under sterile conditions with energy densities (light doses) of 0 to 10 J/cm^2^ at 635 nm (5-ALA-derived PpIX) or 0 to 1.5 J/cm^2^ at 595 nm (HYP) through the cover plate of the 4-well plates. Irradiation was performed with the bare fibre used for laser light transmission. Light was transmitted through an optical diaphragm in order to ensure irradiation with the central part of the laser beam. Exposure times were varied to obtain the various light doses at constant power density (fluence rate) of 20 mW/cm^2^ (5-ALA-derived PpIX) and 10 mW/cm^2^ (HYP). All illumination experiments were monitored with a power meter (model TPM-310, Gentec, Quebec, Canada).

Cell survival was assessed photometrically (NR assay as described above) after an additional growth period of 48 h (37°C, 5% CO_2_). Survival rates were calculated as percentages of the living cells after incubation and treatment by PDT compared to the non-illuminated controls. To compare the response of the different cell lines towards PDT and to compare efficacy of both photosensitizers, 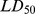-values [lethal light doses required to reduce cell survival to 50% of the controls [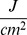]] were calculated from dose response curves.

### Statistical Analysis

For mathematical description of the concentration- and time-dependent HYP and 5-ALA-derived PpIX uptake special compartmental models as described in more general detail in Jacquez were used [29].

#### Concentration-dependent HYP uptake and 5-ALA-derived PpIX fluorescence

The relationship between the concentration of the fluorescent marker 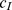 [5-ALA: mM, HYP µM] and intracellular fluorescence 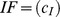, as measured in arbitrary units [a.u.] by FM and FACS, was calculated by the following formula:
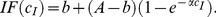
(1)


The parameters were fitted by the nonlinear least square method after logarithmic transformation of the mean observations.

The parameter 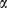
[1/[mM or µM] describes the rate of approach of HYP fluorescence to the asymptote 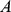 [a.u.]. 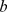
[a.u.] is the autofluorescence, and 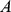 [a.u.] is the asymptote for large concentrations. For both methods, the parameters 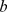 and 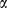 were compared by two-sample t-test, and 95% confidence intervals were calculated.

The percentage of variability which is explained by the model according to the formula
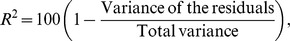
was calculated for each model. The residuals (R) are the differences of the observed values and the values predicted by the model. For some models we calculated relative residuals by taking logarithms. Otherwise we calculated absolute residuals.

#### Time-dependent HYP uptake

For determination of time-dependent HYP uptake by FM and FACS the relation between incubation time (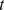) [h] and intracellular fluorescence (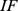) [a.u.] intensity was calculated as follows:
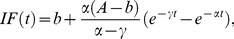
(2)where 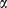 [1/h] is the rate of increase of the fluorescence intensity, 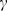 [1/h] is the rate of decrease of the fluorescence intensity (e.g., by photobleaching), 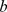 [a.u.] is the autofluorescence, and 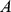 [a.u.] determines the peak fluorescence intensity.

#### Comparison of fluorescence microscopy and flow cytometry

FM and FACS were compared with respect to the rates 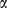 by two-factorial analysis of variance, taking into account the method, the concentration of the photosensitizer, and their interaction. Methods were compared by post hoc t-test.

#### Cellular uptake by temperature

Time-dependent HYP uptake 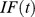 [a.u.] was measured by FACS and fitted by Formula 3a:
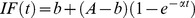
(3a)where 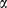 [1/h] is the rate of approach to the asymptote, 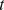 [h] is the incubation time, 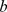
[a.u.] is the autofluorescence, and
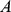
[a.u.] is the asymptote for large times. Formula (3a) is identical to formula (2) for 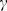 = 0.

Fluorescence intensity is described by model (3a) with 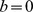 at constant temperature. For temperature change at time 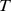 ^*^, the initial increase in fluorescence intensity at low temperatures is described by 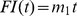 [a.u.].

For times after the temperature switch at time 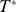, the formula becomes.
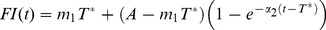
(3b)


The initial slope for 37°C 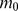 [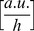
is 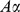. The slope 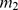 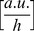 after the switch in temperature from 4°C to 37°C is 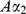.

#### Photocytotoxicity

Relationship between cell survival 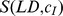 [%] and light dose 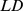
[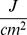] and incubation concentration 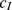 [mM] for 5-ALA-derived PpIX was calculated by:
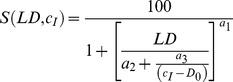
(4)where 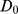 [mM] is the minimal incubation concentration, 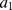 (dimensionless) determines the slope at the point of inflection, 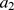
is the asymptotic 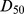
[mM] as 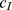 reaches infinity, and 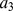 [mM^2^] determines the approach to the asymptotic 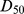. The 95% confidence interval was calculated, and the cell lines were compared by z-test.

The relationship between cell survival 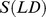 [%] and light dose 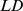
[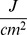] for HYP PDT was expressed by:
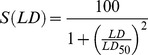
(5)were 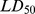 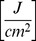
is defined as the light dose needed to inactivate 50% of living cells (lethal light dose 50).

Values of quantitative data are given as median (central tendency) +/− median absolute deviation (MAD, variability) except otherwise noted.

## Results

### Cytotoxicity

Cytotoxicity (in the darkness) of 5-ALA/5-ALA-derived PpIX and HYP was exemplarily determined in D283 Med cells. In the case of 5-ALA/5-ALA-derived PpIX no cytotoxicity was found for concentrations up to 6 mM and an incubation time of 2 h. For higher incubation concentrations, however, this compound exhibited pronounced cytotoxicity resulting in survival rates of 50.1% (MAD ±17.8%) at 9 mM and 17.9% (MAD ±6.6%) at 15 mM after 2h-incubation. In contrast, HYP was not cytotoxic to D283 Med cells up to 20 µM after 2 h incubation.

### Accumulation of 5-ALA-derived PpIX

#### Concentration-dependent 5-ALA-derived PpIX fluorescence

PpIX fluorescence was measured by fluorescence microscopy in D283 Med medulloblastoma cells incubated with 5-ALA at concentrations between 0 and 6 mM for 2 h. Autofluorescence of the cells was assessed using non-incubated controls. Intracellular PpIX fluorescence increased from 204 to 250 a.u., i.e. by about 22% compared with autofluorescence levels, after incubation with 1.2 mM 5-ALA. However, incubation with higher 5-ALA concentrations (up to 6 mM) resulted only in a further 2% increase in PpIX accumulation (see Figure 1).

### Accumulation of HYP

#### Concentration-dependent uptake by FM

Concentration-dependent uptake of HYP was examined in D283 Med, Daoy, and D341 Med cells by fluorescence microscopy. Cells were incubated at HYP concentrations between 0 and 20 µM for 2 h. Figure 2 shows the intracellular accumulation of HYP fitted by Formula (1). For all cell lines, fluorescence intensity increased almost linearly up to 2.5 µM HYP. However, fluorescence increase was less pronounced at higher concentrations. D341 Med and Daoy cells exhibited nearly identical fluorescence levels, whereas that of D283 Med cells was significantly higher.

Figure 3A shows the intracellular HYP accumulation in three independent experiments of D283 Med incubated with different HYP concentrations by FM. Addionally, concentration-dependent HYP uptake was measured also by FACS in D283 Med cells under identical conditions as used in the FM experiment (Fig. 3B). Results obtained with both methods were fitted by Formula (1). HYP fluorescence increased almost linearly up to incubation concentrations of 2,5 µM, whereas fluorescence increase was less pronounced at the higher concentrations (up to 20 µM). As depicted in Figure 3C, both methods led to quite comparable results considering the significant difference in autofluorescence (mean autofluorescence values 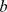: 97.4 a.u. (95% CI: 79.3–119.3) for FM and 5.4 a.u. (95% CI: 4.4–6.5) for FACS with p<10^−4^ according to a z-test). Mean 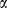 values were calculated according to Formula (1). For FM 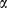
was 0.111 [1/[µM] (95% CI: 0.057–0.174 [1/[µM]) and 0.115 [1/[µM] (95% CI: 0.083–0.150 [1/[µM]) for FACS (p = 0.874).

#### Time-dependent HYP uptake

Time-dependent fluorescence intensity measurements of HYP uptake by FACS and FM are shown together in Figure 4. D283 Med cells were incubated for up to 6 hours with HYP at 2.5, 5, and 10 µM. For an incubation concentration of 10 µM, HYP uptake peaked at 2 h and 4 h for FM and FACS, respectively. For lower HYP concentrations (2.5 and 5 µM) a steady increase of cellular fluorescence was observed.

The parameter 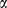, which describes the uptake rate, was estimated for the FACS and FM analyses (Table 1). After Bonferroni adjustment of p-values, there was no significant difference between the two methods at concentrations of 5 and 10 µM (Table 1).

### Temperature Dependent Cellular Uptake

In order to elucidate, whether HYP uptake is an active, energy-dependent process, D283 Med cells were incubated with HYP (2.5 µM) at two different temperatures of 4°C and 37°C. Cellular HYP accumulation was almost negligible within the incubation period at 4°C (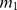 = 1.1). After raising the temperature to 37°C, HYP uptake started to increase (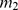 = 9.2). However, intracellular HYP fluorescence did not reach the same uptake rate (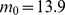) as observed when cells were incubated at 37°C for the entire period (Figure 5). Table 2 summarizes the initial slopes and their confidence intervals for the two conditions. There is a significant difference between the rate of uptake at constant temperature and at delayed temperature increase: The rate of uptake at 37°C for constant temperature is 834 [a.u./h] and 552 [a.u./h] for delayed temperature rise (p<0.0001).

### Photocytotoxicity

#### 5-ALA-derived PpIX

Photocytotoxicity of 5-ALA-derived PpIX was determined in D283 Med cells at up to 6 mM 5-ALA (2h incubation for each) and an illumination wavelength of 635 nm. The light doses in all experiments were 0 to 10 J/cm^2^. As shown in Figure 6A, cell inactivation after incubation with 5-ALA concentrations higher than 0.5 mM depends mainly on the light dose. PDT with a concentration of 0.25 mM 5-ALA or less was dependent on light dose and 5-ALA concentration.

Under the same conditions, PDT was performed in U373 MG glioblastoma cells, incubated with 0 to 1 mM 5-ALA (Figure 6B). In comparison to D283 Med cells, cell inactivation after incubation with 5-ALA concentrations higher than 0.25 mM was mainly dependent on the light dose. To compare 5-ALA-derived PpIX photocytotoxicity in the different cell lines, 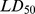
values were calculated according to Formula (4). 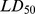 values were significantly different between D283 Med and U373 MG cells at all concentrations (0.1, 0.25, 0.5 and 1.0 mM). 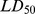 values were lower in D283 Med cells (Table 3) after incubation with 0.5 and 1 mM 5-ALA, i.e. medulloblastoma cells were more sensitive to PDT.

#### Photocytotoxicity of HYP

The photocytotoxicity of HYP in D283 Med cells was determined at a noncytotoxic concentration (2.5 µM), an incubation time of 2 h, and an illumination wavelength of 595 nm; the light doses were 0 to 1 J/cm^2^. The 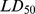 value of HYP was 0.47 J/cm^2^ (95% CI 0.426–0.515). Cell survival was fitted by Formula (5) as depicted in Figure 7.

## Discussion

To improve the prognosis of medulloblastoma patients, new therapeutic strategies are necessary. In treating medulloblastomas, response to adjuvant therapy is amended by increasing the extension of resection. Thus, intraoperative visualization might be a suitable option. 5-ALA-derived PpIX has been successfully applied for the intraoperative visualization of malignant brain tumors improving the prognosis of patients [4].

HYP, a naturally occurring photosensitizer, has many advantages compared to other photosensitizers, e.g. 5-ALA. Recent *in vitro* and *in vivo* studies have demonstrated high efficacy of HYP in PDT for various tumors [26], [30]–[33]. Additionally, HYP inhibits cell proliferation and signal transduction, also in the dark [34], [35]. No data on the visualization and PDT of medulloblastomas using HYP exist, prompting us to investigate this and compare it with 5-ALA-derived PpIX.

### Accumulation of 5-ALA-derived PpIX

By FM, D283 Med cells showed a concentration-dependent accumulation of PpIX. However, fluorescence increase was about 20% as compared to autofluorescence after an incubation period of 2 h with concentrations up to 1.2 mM 5-ALA. Other groups have reported disparate results with regard to PpIX fluorescence after incubation with 5-ALA *in vitro*. Stummer incubated C6 glioma cells *in vitro* with 1 mM 5-ALA, observing a linear rise in PpIX fluorescence from 5 to 85 min by fluorescence spectroscopy [36]. Carre et al. noted increasing PpIX synthesis in C6 glioma cells but reported high intercell variability by confocal laser scanning microspectrofluorometry, exciting cell samples at 488 nm [37].

Great differences were seen in *ex vivo* glioblastomas after *in vitro* incubation with 5-ALA. PpIX synthesis ranged from undetectable to high [38]. Moan et al. noted a time-dependent linear increase in PpIX after *in vitro* incubation with 1 mM 5-ALA for 0 to 8 h [39]. Wyss-Desserich et al. observed up to a 4-fold increase in fluorescence intensity in malignant cells versus normal cells incubated with 0.06 mM up to 60 mM 5-ALA for 3, 6, and 24 h [40]. Steinbach et al. demonstrated by FACS measurements that fluorescence of 5-ALA-derived PpIX increased linearly up to 4 h in human bladder carcinomas when incubated *in vitro* with 0.18 to 1.8 mM 5-ALA. Sailer et al. noted different amounts of PpIX accumulation as well as a varying intracellular localization of PpIX in different cell types, implicating disparate responses of tumours of different origin towards PDT [41]. According to Amo et al. PDT-induced cell death seems to occur predominantly via apoptosis through 5-ALA induced PpIX in mitochondria [42].

The small amount of PpIX accumulation in medulloblastoma cells may be explained by the large nucleus to cytoplasm ratio that may limit the primarily perinuclear production of PpIX [41], [43]. Another explanation for the lack of increase in fluorescence intensity might be the generation of non-fluorescent aggregates. According to Schneckenburger et al., porphyrins tend to form aggregates with poor fluorescence in solution as well as within cells [44]. However, the presented data demonstrates that PpIX production after 5-ALA incubation is quite comparable in medulloblastoma and glioblastoma cells.

### HYP Uptake in Medulloblastomas

All 3 medulloblastoma cell lines showed time- and concentration-dependent HYP accumulation, based on fluorescence intensity measurements by FM and FACS. High incubation concentration of HYP (10 µM) led to a rapid increase in fluorescence within 2 h. For longer incubation times up to 6 h, HYP fluorescence declined by about 15% at high HYP concentrations. Incubation with lower HYP concentrations (e.g. 2.5 µM) reached saturation not until 6 hours, which agrees with findings from our earlier studies in glioblastoma cell lines [26], [45]. Similarly to the presented data, Ali et al. examined HYP uptake in nasopharyngeal carcinoma cells (CNE2 and TW0–1) incubating them with 1.25 µM (CNE2) and 2.5 µM HYP (TW0–1). HYP fluorescence rose during the first 2 to 3 h and decreased after 4 h [26], [46]. This phenomenon might be due to the aggregation of PS molecules at higher concentrations, resulting in lower fluorescence [44]. Further on the accumulation of HYP in the tumor, compared to normal tissue, depends whether HYP is dissolved in a solution or in coarse aggregates. HYP soluted in a mixture of polyethylene glycol, DMSO and water showed a superior tumour to tissue relation compared to HYP suspended as coarse aggregates [47]. Not only the incubation concentration but also the uptake time has some influence to the HYP aggregation and by this to the phototoxicity of HYP [48]. This is not only due to aggregation processes but also to dynamics of the intracellular traffic [49].

So far, the mechanism(s) by which HYP is taken up is unknown. The presented data demonstrate a temperature-dependent uptake of HYP in a medulloblastoma cell line. Quite similar results have been obtained previously for glioblastoma cells [26]. With this experiment it is not possible to investigate the transport mechanism(s) of HYP in detail, i.e. to distinguish between micropinocytosis or receptor-mediated transport mechanisms. However, it is appropriate to distinguish whether HYP is predominantly taken up by an active, energy-dependent process or by passive diffusion (energy-independent). Analyzing fluorescence images, Uzdensky et al. found no difference in HYP staining patterns in WiDr cells (human colon carcinoma cell line) after a 1 h incubation at 4°C or room temperature, concluding that the uptake occurred by diffusion [50]. However, the procedure of the experiment has not been described in detail. Active uptake of HYP has also been observed in Caco-2 cell monolayers, a model that is used to study intestinal absorption. Transcellular transport of HYP at 37°C was significantly higher compared with that at 4°C [51].

### Photodynamic Inactivation

5-ALA-derived PpIX showed photodynamic inactivation of U373 MG and D283 Med cells upon irradiation at 635 nm with light doses up to 10 J/cm^2^ after 2 h incubation with 5-ALA at a non-cytotoxic concentration (0.5 to 6 mM). The light dose that was needed to inactivate 50% of cells, also named 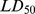 value, was significantly lower in the medulloblastoma cell line as compared to the glioblastoma cell line applying incubation concentrations of 0.5 and 1 mM 5-ALA. However, photodynamic inactivation of D283 Med cells (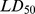 44.6 J/cm^2^) was less efficient as compared to U373 MG cells (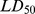 12.6 J/cm^2^) at a lower 5-ALA incubation concentration (0.1 and 0.25 mM). Riesenberg et al. also observed disparate responses of several human bladder carcinoma cell lines *in vitro* to PDT with 0.15 and 0.6 mM 5-ALA and light doses ranging from 15 to 100 J/cm^2^ [52].

In D283 Med cells, HYP exhibited high photocytotoxicity. HYP-PDT of D283 Med cells (2 h/2.5 µM) irradiated at 595 nm with light doses of 1 J/cm^2^ resulted in >90% cell death and an 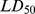 value of 0.47 J/cm^2^ was calculated. For glioblastoma cell lines (U373 MG, LN229, and T98G), 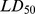 values varied between 0.15 and 0.22 J/cm^2^ after 2h-incubation with 2.5 µM HYP as reported previously [26]. These results demonstrated HYP is much more efficient for photodynamic treatment of medulloblastomas and gliomas *in vitro* as compared to 5-ALA-derived PpIX (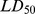 of 6.5 J/cm^2^ at 0.25 mM). The high photodynamic efficacy of HYP is most likely due to its high triplet quantum yield and efficient induction of ROS [16], [17], [53]. High efficacy of HYP-PDT may at least compensate the lower penetration depth of 595 nm light into tissue as compared to the most effective wavelength used in 5-ALA-PDT (635 nm). However, more clarity can be gained only by *in vivo* experiments. For this purpose, orthotopic *in vivo* models are indispensable. Recently reported *in vivo* models, for instance the topic application of HYP and 5-ALA in a skin tumor model [54], are not sufficient to predict the therapeutic effects in brain tumors, where the blood brain barrier has also to be considered.

### Conclusions

Medulloblastoma cell lines exhibited significant time- and concentration-dependent HYP accumulation (5-fold over autofluorescence levels) and HYP is probably taken up by an energy dependent mechanism. The high photocytotoxicity of HYP as compared to 5-ALA-derived PpIX (8–13-fold lower 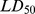 values) is notable. Therefore, HYP might be a promising molecule for intraoperative visualization and photodynamic treatment of medulloblastomas. In case that these promising *in vitro* data are confirmed by *in vivo* experiments, HYP has the potential to widen the armamentarium of medulloblastoma treatment options in primary treatment and especially as an alternative treatment option in cases of tumor relapse after extensive radiation and chemotherapy.
